# 724. Prevalence and Risk Factors for Carbapenem Resistant Enterobacterales Colonization on Admission to Two Intensive Care Units in India

**DOI:** 10.1093/ofid/ofad500.785

**Published:** 2023-11-27

**Authors:** Armaghan-e-Rehman Mansoor, Fabia Edathadathil, Devendhu Suresh, Yathu Krishna, Anu George, Jacaranda van Rheenen, Dorothy Sinclair, Margaret A Olsen, Anil K Vasudevan, Jennie H Kwon, Surbhi Leekha, Sanjeev Singh, David K Warren, Sumanth Gandra

**Affiliations:** Washington University in St. Louis, Lexington, KY; Amrita Institute of Medical Sciences and Research Center, kochi, Kerala, India; Amrita Institute of Medical Sciences, Kochi, Kerala, India; Amrita Institute of Medical Sciences, Kochi, Kerala, India; Amrita Institute of Medical Sciences, Kochi, Kochi, Kerala, India; Washington University School of Medicine in St. Louis, Saint Louis, Missouri; Washington University School of Medicine in St. Louis, Saint Louis, Missouri; Washington University School of Medicine in St. Louis, Saint Louis, Missouri; Amrita Institute of Medical Sciences, Kochi, Kerala, India; Washington University - School of Medicine, St. Louis, MO; University of Maryland School of Medicine, Baltimore, MD; Amrita Institute of Medical Sciences, Kochi, Kochi, Kerala, India; Washington University School of Medicine in St. Louis, Saint Louis, Missouri; Washington University School of Medicine in St. Louis, Saint Louis, Missouri

## Abstract

**Background:**

Carbapenem-resistant *Enterobacterales* (CRE) infections are endemic in Indian hospitals however, studies examining intestinal colonization of CRE on admission to intensive care units (ICUs) are very limited. This study examines the prevalence and risk factors for CRE colonization on admission to two ICUs in a tertiary-care hospital in India.

**Methods:**

Peri-rectal swabs were collected from consented patients prospectively enrolled between December 16, 2022 and March 31, 2023 in the medical ICU (MICU) and surgical ICU (SICU). Swabs were plated on selective agar (CHROMagar^TM^mSuperCARBA^TM^) within 24 hours of collection for CRE isolation. Identification and susceptibility of presumed CRE isolates were confirmed by VITEK2. The modified carbapenem inactivation (mCIM) test and the EDTA carbapenem inactivation method (eCIM) were used to assess carbapenemase production. Information on CRE colonization risk factors (hospitalization and ICU stay in last one year, transfer from outside hospital, long-term hemodialysis) was collected.

**Results:**

A total of 453 patients were admitted to both ICUs during the study period and 158 patients gave consent. Overall CRE prevalence among 158 patients was 54% (86/158). Peri-rectal swab was obtained within 48 hours of admission for 78 patients (Figure 1), and CRE prevalence in this cohort was 42% [33/78], with significantly higher prevalence noted among patients in the MICU compared to SICU (63% [17/27] vs 31% [16/51], p=0.007). Hospitalization, or ICU admission within the last 1 year, and long-term dialysis were significantly associated with CRE colonization (Table 1).

Among 33 patients with CRE colonization on admission, the most common CRE isolated was *Klebsiella pneumoniae* (59% [20/34 isolates]) followed by *Escherichia coli* (38% [13/34 isolates]). Among the 34 CRE isolates, 19 were metallo-beta-lactamase producers and 15 were serine carbapenemase producers.

**Figure:**
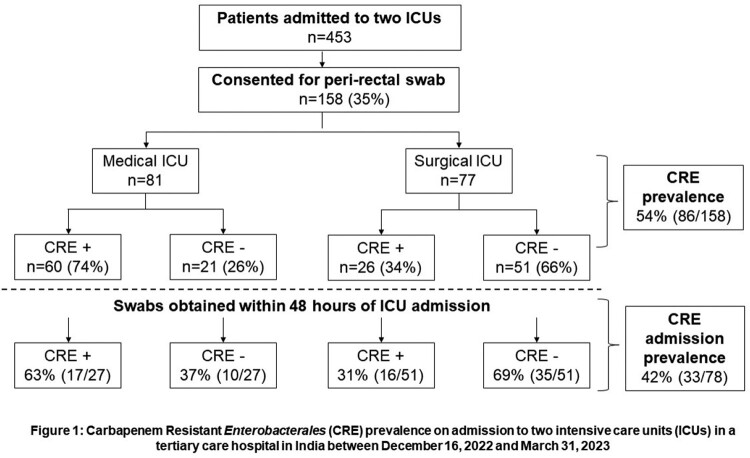

Table 1

**Figure:**
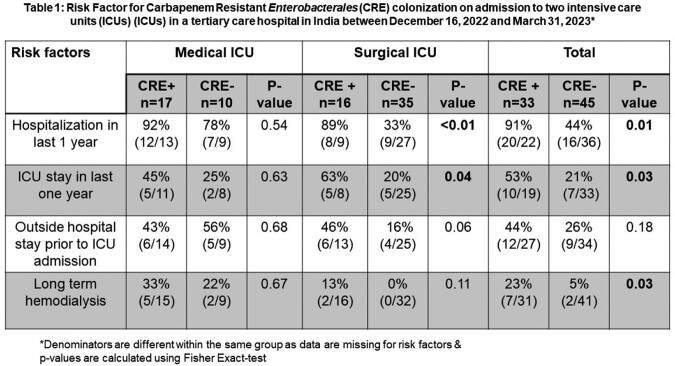

**Conclusion:**

High prevalence of CRE colonization on admission was observed in the two ICUs, with prior hospitalization significantly associated with CRE colonization. Significantly higher proportion of CRE colonization was seen in the MICU as compared to SICU. Future studies should examine the risk factors associated with CRE acquisition during hospitalization to design interventions.

**Disclosures:**

**All Authors**: No reported disclosures

